# Specific Irreversible Cell-Cycle Arrest and Depletion of Cancer Cells Obtained by Combining Curcumin and the Flavonoids Quercetin and Fisetin

**DOI:** 10.3390/genes13071125

**Published:** 2022-06-23

**Authors:** Viviana Barra, Roberta Flavia Chiavetta, Simona Titoli, Ivana Maria Provenzano, Pietro Salvatore Carollo, Aldo Di Leonardo

**Affiliations:** Department of Biological, Chemical and Pharmaceutical Sciences and Technologies, University of Palermo, 90128 Palermo, Italy; roberta.chiavetta@unipa.it (R.F.C.); simona.titoli@unipa.it (S.T.); ivanamaria.provenzano@community.unipa.it (I.M.P.); pietrosalvatore.carollo@community.unipa.it (P.S.C.)

**Keywords:** senescence, curcumin, senolytics, heterochromatin, DNA methylation, H3K9 trimethylation, SAHF, fisetin, quercetin

## Abstract

**Background:** Induced senescence could be exploited to selectively counteract the proliferation of cancer cells and target them for senolysis. We examined the cellular senescence induced by curcumin and whether it could be targeted by fisetin and quercetin, flavonoids with senolytic activity. **Methods:** Cell-cycle profiles, chromosome number and structure, and heterochromatin markers were evaluated via flow cytometry, metaphase spreads, and immunofluorescence, respectively. The activation of p21^waf1/cip1^ was assessed via RT-qPCR and immunoblotting. Senescent cells were detected via SA-β-Galactosidase staining. **Results:** We report that curcumin treatment specifically triggers senescence in cancer cells by inducing mitotic slippage and DNA damage. We show that curcumin-induced senescence is p21^waf1/cip1^-dependent and characterized by heterochromatin loss. Finally, we found that flavonoids clear curcumin-induced senescent cancer cells. **Conclusions:** Our findings expand the characterization of curcumin-induced cellular senescence in cancer cells and lay the foundation for the combination of curcumin and flavonoids as a possible anti-cancer therapy.

## 1. Introduction

Halting cancer cells’ proliferation has always been a tough challenge, because its main difficulties are related to the high molecular heterogeneity of tumors and their altered genetic and epigenetic background. In particular, cancer cells frequently show an impairment of the pathways that normally arrest cell proliferation in the presence of DNA damage, genetic alterations, and genomic instability [1]; thus, forcing the reactivation of these pathways, such as those leading to cellular senescence, could be a valid strategy to counteract cancer cell proliferation.

Cellular senescence is defined as the irreversible arrest of the cell cycle [2]. It was originally described by Leonard Hayflick, who noticed that cultured normal cells reach a limit on cell divisions. This limited capacity to divide depends on telomere length, which shortens with every cell division. Telomere shortening, in turn, activates a persistent DNA damage response, which leads to permanent cell-cycle arrest, known as cell senescence [3,4]. The activation of such a response avoids the propagation of DNA/chromosome damage and preserves the genetic information. In addition, other stress signals (ROS, radiations, other DNA damages, and oncogenes activation), both endogenous and exogenous, can trigger cellular senescence; this, in turn, acts as a protective mechanism to avoid the spread of altered cells [5].

A senescent cell does not divide, but it is still metabolically active. It synthesizes and produces mediators that make it highly recognizable and that serve to maintain and propagate the senescent phenotype. For example, p21^waf1/cip1^, or p16INK4a, and β-galactosidase enzymes are usually highly active in senescent cells; for this reason, they are frequently used as senescence markers [6]. Moreover, lipofuscin granules, composed of carbohydrates and proteins derived from cell catabolic activity, accumulate in senescent cells and are visible as dark granules [7]. Lastly, especially during late senescence, senescent cells produce specific inflammatory interleukins, and cytokines, with both autocrine and paracrine actions; their main function is to maintain and extend the senescent phenotype to form a barrier against the damaged cell [8]. The ensemble of these proteins is called the Senescence-Associated Secretory Phenotype (SASP); it is one of the features most frequently associated with senescent cells, despite that fact that its components vary according to the type of senescence induced and the stage of senescence reached. Senescence establishment is a long, organized process in which every phase, from the earliest to the latest, is characterized by the production of specific mediators [8]. In addition to these molecular changes, senescent cells are characterized by morphological changes, namely an enlarged and flattened cell shape and alterations to the nuclear membrane [9]. It is also worth mentioning that senescent cells undergo chromatin reorganization. In dividing cells, heterochromatin is mainly compartmentalized at the nuclear periphery, while during senescence—similarly to the aging process—heterochromatin is progressively lost; however, dense foci of heterochromatin (Senescence-Associated Heterochromatin Foci—SAHF) are often visible in the nucleoplasm [10,11,12]. Related to this phenomenon, epigenetic markers are also rearranged, with H3K9me2/3 and DNA methylation progressively lost globally, and especially at repetitive sequences [10,13]; conversely enrichment of H3K9me3 and macroH2A occurs at the SAHF [14]. However, it has been observed that SAHF are not present in all senescent cells, as they most probably depend on cell type and stimulus [15]. Indeed, SAHF seem to be more prominent in oncogene-induced senescence (OIS) than in replicative senescence [11,16]. Moreover, cells from Hutchinson–Gilford progeria syndrome patients, who undergo pathological accelerated aging, are characterized by a reduction in heterochromatin, but do not form SAHF. This suggests the existence of a two-step mechanism to first decondense chromatin and then rearrange the heterochromatin in the SAHF [17].

Clearly, senescence induction in cancer cells can be a powerful strategy in counteracting tumor spread. In particular, the reactivation of senescence-inducing pathways—specifically in cancer cells, and not in non-cancerous immortalized cells—is an important goal to generate anti-cancer therapies that could be safer for patients. It has recently been shown that some molecules display pronounced anti-cancer activity by triggering senescence in cancer cells. For example, the natural polyphenol curcumin, derived from *Curcuma longa*, has been shown to induce senescence in HCT116 cancer cells by causing cell-cycle arrest in the G2/M phase [18,19,20,21]. Herein, we investigate the mechanism/s underlying curcumin-mediated senescence, confirming its more specific action against cancer cells. We analyze several markers of senescence and look at the presence of DNA damage. Lastly, we show that induced senescent cancer cells can also be specifically cleared by fisetin and quercetin. These two flavonoids, known for their broad anti-inflammatory action, can act in cells both as senescence inducers and senolytic drugs, depending on the dose [22]. For the latter function, flavonoids have been shown to play an important role in counteracting aging by removing senescent cells [23,24]. Coupling senolysis, described as the induction of apoptosis in senescent cells, with senescent inducers such as curcumin, can be a potential strategy in the development of anti-cancer therapies; this might allow the selective elimination of senescent cancer cells, without affecting the non-cancerous cells of the organism.

## 2. Materials and Methods

### 2.1. Cells and Cell Culture

HCT116 cells with the MIN phenotype (near-diploid) (kindly provided by Dr B. Vogelstein, John Hopkins University, Baltimore, MD), MCF-7, and RPE-1 hTERT cells were cultured in DMEM with 10% FBS (Corning, catalogue number 35-010-CV), 100 U/mL penicillin, and 0.1 mg/mL streptomycin. Cells were cultured in a humidified atmosphere with 5% CO_2_ at 37 °C. Cells were usually divided when they reached a confluency of 90–100%. Cells were treated with 10 µM of curcumin for 24 h and then released into fresh medium. Additional treatment for 72 h with 5 or 10 μM of fisetin and quercetin was also administered. All of the used molecules (curcumin, fisetin, quercetin) (kindly provided by Prof. Riela and Dr. Massaro, University of Palermo) were dissolved in DMSO. DMSO treatment was used as a control. The DMSO percentage in both treated and control cells was lower than 0.1% of the total volume. 

### 2.2. Cell-Cycle Analysis

Cell-cycle profiles of asynchronously growing cells were analyzed via flow cytometry. The DNA content was determined by staining cells with Propidium Iodide (PI, Merk-Sigma-Aldrich, Milan, Italy), a DNA dye that binds the DNA in a stoichiometric way. The DNA amount enables the detection of cells in each phase of the cell cycle: the G0/G1, S, and G2/M phases. Aliquots of 10^6^ cells were obtained via trypsinization and collected in cold Phosphate–Buffered Solution (PBS). Cells were then harvested via centrifugation (10 min at 1000 rpm), washed with PBS, and incubated in the dark in PBS containing 20 μg/mL PI and 40 μg/mL RNase, for 30 min, at room temperature. Samples were then subjected to fluorescence-activated cell-sorting analysis using a FACSCanto flow cytometer (Becton Dickinson Biosciences). At least 20,000 cells were analyzed for each sample using the FACSDiva software, Kaluza software and FlowJo software. Discrimination of cell doublets from singlets was carried out by plotting the DNA dye (PI) channel width versus its area in a dot-plot graph as previously described [25]. Gating was carried out by distinguishing 2n- and 4n-content cells (G1 and G2/M phase, respectively) on the base of DNA content.

### 2.3. SA-β-Gal Staining

Cellular senescence was evaluated using an SA-β-Gal assay. Cells were cultured in triplicate in an MW12 plate and, after medium removal and washes with PBS, fixed on the day of the analysis with 4% PFA (paraformaldehyde, pre-heated at 37 °C) for 5 min at room temperature. Cells were then washed twice with PBS for 5 min, and incubated for 24 h at 37 °C with a staining solution containing the following in 40 mM citric acid/sodium phosphate solution, pH 6.0:

- X-Gal (5-bromo-4-chloro-3-indolyl-β-d-galactopyranoside; Promega, Milan, Italy, catalogue number V3941) 0.1%;
- 5 mM of potassium ferrocyanide and 5 mM of potassium ferricyanide;
- 150 mM Sodium chloride;
- 2 mM Magnesium chloride.

The staining solution was removed the next day, and cells were washed twice with distilled water; then, they were observed under a transmitted light microscope with a 20× objective. The percentage of senescent cells was evaluated on 600 cells for HCT116 and 100 cells for RPE-1 hTERT. At least two independent experiments with two technical replicas were performed. 

### 2.4. Immunofluorescence

#### 2.4.1. γH2AX

Cells were seeded the day before the treatment on glass coverslips, which were placed on the bottoms of wells of an MW12 plate. Cells were treated at a 40% confluence with curcumin 10 µM. On the day of the analysis, cells were washed twice with PBS and fixed with ice-cold methanol for 5 min at room temperature, or until evaporation. After two washes with PBS, cells were permeabilized with 0.01% Triton-X diluted in mQ water for 10 min at room temperature; then, they were washed again and blocked with 0.1% BSA (Bovine Serum Albumin) for 30 min at room temperature. Antibody labeling was carried out by diluting antibodies in 0.1% BSA. The primary antibody against γH2AX (Anti-phospho-Histone H2A.X (Ser139) Antibody, clone JBW301, Upstate^®®^, from mouse; catalogue number 05-636) was diluted at 1:200 and incubated overnight at 4 °C. The secondary antibody (Goat Anti-Mouse IgG H&L (Alexa Fluor^®®^ 488), ab150113, Abcam, Cambridge, UK) was used at 1:500 and left for 1 h at room temperature. Each labeling was followed by PBS washes. DAPI (1 μg/mL) was added before observation with a Zeiss Axioskop microscope. 

#### 2.4.2. 5-Methylcytosine

For 5-Methylcytosine staining cells were grown onto coverslips, then washed with 0.1% Tween-20 in PBS and fixed for 10 min in freshly prepared 4% formaldehyde in PBS. Cells were permeabilized with 0.5% Triton X-100 in PBS for 30 min and treated with 2M HCl for 30 min at 25 °C. After extensive washing with 0.1% Tween-20 in PBS, cells were blocked for 1 h in 2% BSA in 0.1% Tween-20 in PBS; then, they were incubated with 1:500 anti-5MC antibody (5-Methylcytosine Monoclonal Antibody, Epigentek, Farmingdale, NY, USA, from mouse, clone 33D3; catalogue number A-1014) in 0.1% Tween-20 in PBS, overnight, at 4 °C; and subsequently, after washing, they were labelled with 1:500 anti-mouse Cy3-conjugated in 0.1% Tween-20 in PBS, for 1 h, at 37 °C. DNA was stained with DAPI (4′,6-diamidino-2-phenylindole) (1 µg/mL) for 5 min at room temperature, and cells were imaged with a Zeiss Axioskop microscope. The experiment was repeated three times, and at least 50 nuclei/sample were quantified for each experiment.

#### 2.4.3. H3K9me3

For H3K9me3 staining, cells were grown onto coverslips, then washed with PBS and fixed for 10 min in freshly prepared 4% formaldehyde in PBS. Cells were permeabilized with 0.5% Triton X-100 in PBS for 10 min. After extensive washing with PBS, cells were blocked for 1 h in 5% BSA and then incubated with 1:100 anti-H3K9me3 antibody (Anti-trimethyl Histone H3 (Lys9), clone 6F12-H4, from mouse; Merck, Milan, Italy; catalogue number 05-1242-S) in 5% BSA, for 1 h, at room temperature; after five washes, they were labelled with 1:1000 anti-mouse Cy3-conjugated in 5% BSA, for 1 h, at room temperature. DNA was stained with DAPI (1 µg/mL) for 5 min at room temperature, and cells were imaged with a Zeiss Axioskop microscope. The experiment was repeated three times, and at least 50 nuclei/sample were quantified for each experiment.

#### 2.4.4. MPM-2

For MPM2 staining, the manufacturer’s instructions for MPM2 antibody were followed. Briefly, cells were grown onto coverslips, then washed with PBS and fixed for 10 min in freshly prepared 4% formaldehyde in PBS. Cells were permeabilized with ice-cold methanol for 5 min on ice. After extensive washing with PBS, cells were blocked for 1 h in 1% BSA at room temperature and then incubated with 1:100 of anti-phospho-Ser/Thr-Pro, MPM2 (mitotic protein monoclonal #2, from mouse; Merck, Milan, Italy, catalogue number 05-368) in 1% BSA in PBS, overnight, at 4 °C. Subsequently, after washing, cells were labeled with 1:1000 anti-mouse Cy3-conjugated (Anti-mouse IgG (H + L), Cy3 conjugated secondary antibody, Jackson ImmunoResearch; catalogue number 715-225-150) in PBS, for 1.5 h, at 37 °C. DNA was stained with DAPI (1 µg/mL) for 5 min at room temperature, and cells were imaged with a Zeiss Axioskop microscope. The experiment was repeated three times, and at least 100 nuclei/sample were quantified for each experiment.

### 2.5. Image Analysis

5-Methylcytosine and H3K9me3 signals were quantified by using a FIJI-based automated system. A mask of the nuclei was obtained by thresholding the DAPI channel, and individual nuclei were detected using the Analyze Particles function. Five 15 × 15-pixel circles were drawn outside the nuclei (marked by DAPI staining) and the means of the integrated signal was calculated (background). The integrated signal intensity of each nucleus was then calculated by subtracting the background as previously performed in [26].

MPM2 staining was analyzed in FIJI as follows: cells were manually counted by using the DAPI channel. All of the images with MPM2 signal were background-subtracted, and then merged with the DAPI channel to obtain the right match between DNA and MPM2. Then, MPM2 spots were manually counted for every single nucleus.

Positivity to phospho-γH2AX staining was considered according to the presence of spots inside cells’ nuclei. Diffuse fluorescence was considered as a background signal. A total of 100 nuclei and 60 nuclei were counted for HCT116 cells and RPE-1 hTERT cells, respectively.

### 2.6. RT-qPCR

RNA was extracted from at least 1 × 10^6^ cells using the ReliaPrep^TM^ RNA Cell Miniprep System (Promega, Milan, Italy). RNA was quantified using Nanodrop^TM^ and loaded on 1% agarose gel to verify its integrity. An amount of 1 μg of RNA was reverse-transcribed in cDNA using the High-Capacity cDNA Reverse Transcription Kit (Thermo Fisher Scientific, Milan, Italy) with the following conditions, according to the manufacturer’s instructions:

**Step 1****Step 2****Step 3****Step 4**Temperature (°C)2537854Time10 min120 min5 min∞

RT-qPCR was performed using Applied Biosystems 7300 Real-Time PCR System Software. Each sample was analyzed in triplicate (50 ng of cDNA/replicate), with a final volume of 25 μL for each replicate. The reaction mix was prepared as follows (the indicated volumes and concentrations are referred to as one replicate):

- 12.5 μL 1× Master Mix SyBR Green (Applied Biosystems, catalogue number: 4367659);
- 2 μM Forward- and Reverse-primer mix;
- RNase-/DNase-free H_2_O.

Gene expression was quantified by comparing the 2^−ΔΔCt^ of each gene to the one of the endogenous gene GAPDH (Forward primer: 5′-CTCATGACCACAGTCCATGCC-3′; Reverse primer: 5′-CAATCCACAGTCTTCTGGGT-3′) as previously performed in [27]. P21^waf1/cip1^ sequence was targeted with the Forward primer 5′-CTGGAGACTCTCAGGGTCGA-3′ and the Reverse primer 5′-CGGATTAGGGCTTCCTCTTG-3′.

### 2.7. Metaphase Spread

Ploidy of the cells was analyzed using a metaphase spread assay. Cells were seeded to be at 70% of confluency on the day of the analysis on microscope slides, to avoid artifacts due to drip. On the day of the analysis, cells were treated with Colcemid (0.2 μg/mL, Merck–Sigma-Aldrich, Milan, Italy) for 2 h. After one wash in PBS, cells were incubated with 75 mM KCl solution for 20 min at 37 °C, to induce swelling. Nuclei were then fixed with ice-cold fixative (3:1 methanol:glacial acetic acid) and stained with 5% Giemsa staining solution for 8 min. Microscope slides were then rinsed with distilled H_2_O before observation with 20× objective (for the search of metaphases and the calculation of mitotic index), and with the 63× and 100× for chromosome analysis and chromosome-break evaluation, as previously performed in [27]. 

For each sample, at least 20 metaphases were analyzed. The experiment was repeated 4 times.

### 2.8. Western Blot

Cells were lysed with 1× Laemmli Buffer and the proteins denatured at 95 °C for 10 min. An amount of 20 µL of the samples were loaded together with a protein ladder marker in a 10% polyacrylamide gel. The SDS-PAGE gel was run in a Mini-PROTEAN System (Bio-Rad, Italy), with Tris-Glycin pH 8.3 running buffer (0.025 M Trizma base, 0.192 M glycin and 0.1% SDS), firstly at 50 Volts, and then at 80 Volts for the rest of the run (90 min in total). The proteins were then transferred onto a PVDF membrane, and pre-activated in methanol, for 90 min, with constant voltage (V = 100), on ice. After blocking with 5% skimmed milk for 1 h, proteins were probed with a solution of 1:1000 anti-p21^waf1^ (Santa Cruz Biotechnology, catalogue number sc-6246); 1:500 anti-γH2AX (Anti-phospho-Histone H2A.X (Ser139) Antibody, clone JBW301, Upstate^®®^, from mouse; catalogue number 05-636); 1:2000 anti-p53 (Santa Cruz Biotechnology); 1:10,000 anti-β-Tubulin (Sigma-Aldrich, catalogue number T4026); and 1:5000 anti-actin (Thermo Fisher Scientific, Milan, Italy), in 0.5% skimmed milk, and incubated while agitating for 1 h, at room temperature. Incubation with anti-Mouse-HRP secondary antibody (Thermo Fisher Scientific, Milan, Italy) followed, for 1 h at room temperature. Protein bands were revealed using Chemidoc XRS+ 60 (Bio-Rad, Italy) following SuperSignal West Femto Maximum Sensitivity Substrate (Thermo Fisher Scientific, Milan, Italy) incubation. Bands were quantified using ImageJ software as follows: A box was drawn around the bands, including all of the intensity of the bands, with a minimal amount of surrounding background. In the immediate surrounding of each band, an additional region of interest was drawn to designate the background region. The signal within the boxes was quantified. The value of background box was then subtracted from all of the bands’ boxes. To perform normalization, the control sample was used as the reference. The ratio of the signals of the housekeeping protein in each lane, using the reference lane as the numerator, yielded the difference in sample load between the reference and the other samples. This normalization factor was then multiplied by the protein signal for each sample. The mean of at least three replicates was represented in the graphs.

### 2.9. SAHF Staining

Cells were stained as previously reported by Aird and Zhang [28]. Briefly, cells were seeded onto glass coverslips for the duration of the treatment, then washed with PBS and fixed for 10 min in freshly prepared 4% formaldehyde in PBS. After extensive washing with PBS, cells were permeabilized by incubating coverslips in 0.2% Triton X-100 in PBS for 5 min at room temperature and then blocked for 5 min in 3% BSA at room temperature. DNA was stained with DAPI (0.15 µg/mL) in 3% BSA for 3 min at room temperature. Cells were extensively washed with PBS, then imaged with a Zeiss Axioskop microscope. The experiment was repeated twice, and at least 100 nuclei/sample were observed.

### 2.10. AO/EB Assay

HCT116 cells were seeded in a 60 mm Petri dish to reach the 40% of confluency on the day of curcumin treatment. At the end of senolytics treatment, cells were trypsinized, and then collected in cold PBS. Culture media were collected too. Then, cells were centrifuged for 5 min at 1000 rpm; the pellet was resuspended, washed once with PBS, and centrifuged for 5 min at 1000 rpm. Cells were then resuspended in 270 µL of a mix containing AO/EB double-staining solution (1×) and PBS, and the mix was poured on glass coverslips for microscopic observation. A total of 100 cells were counted to quantify each condition.

### 2.11. Clonogenic Assay

1000 HCT116 cells were seeded on 6-well plates, and at the end of treatments with curcumin and senolytics, colonies were fixed for 10 min in methanol. After washing with PBS, cells were stained for 10 min using a crystal violet staining solution (1% crystal violet, 20% EtOH). After washing with PBS, cells were left to dry upside-down, and then images were taken with a scanner.

### 2.12. Trypan Blue Staining

Cell samples were diluted 1:1 in 0.4% Trypan blue solution. Then, cells were counted under a microscope in three 1 × 1 mm squares of a Burker chamber, to determine the average number of cells per square (total cell number and Trypan Blue positive cell number); this was multiplied by 10^4^ to obtain the number of cells per milliliter, and corrected for the dilution.

## 3. Results

### 3.1. Curcumin Leads Cancer Cells and Not Non-Cancerous Immortalized Cells to Senescence

**Figure 1:**
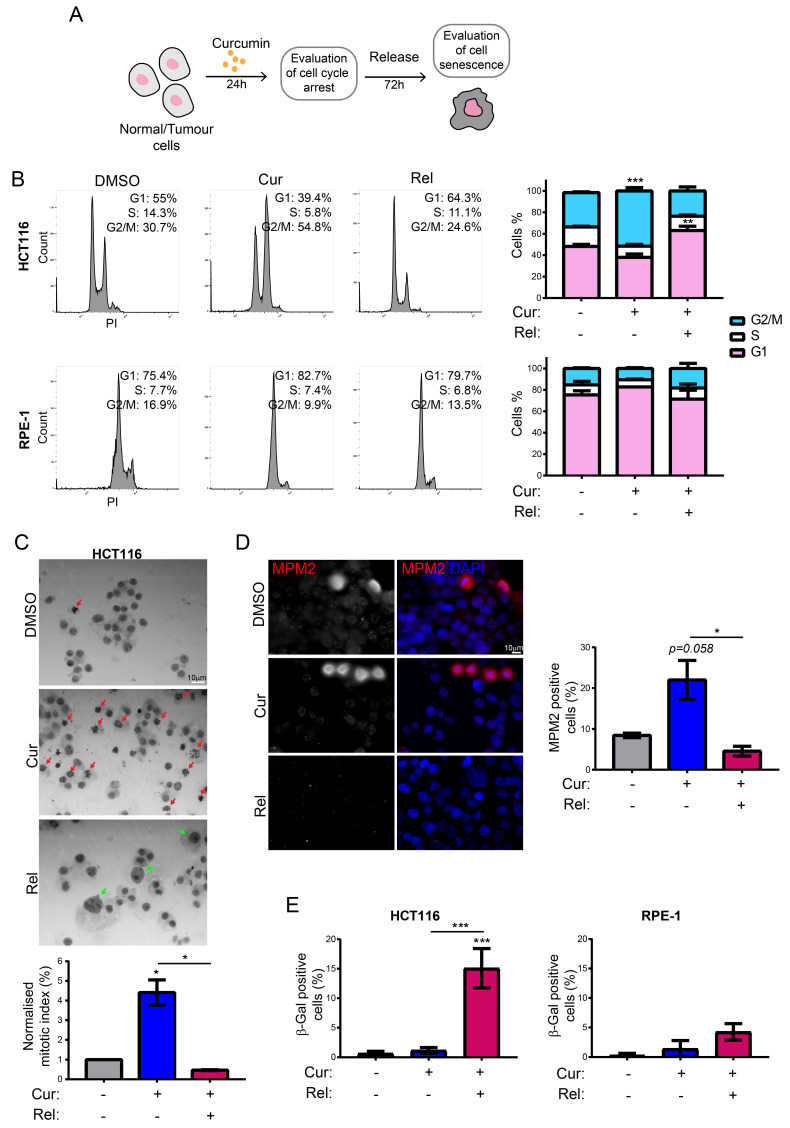
Curcumin treatment selectively induces cell senescence in cancer cells following exit from cell-cycle arrest in mitosis: (**A**) Schematics of the experiment workflow; (**B**) representative flow-cytometric profiles showing the distribution throughout the cell cycle of propidium iodide-labeled HCT116 and RPE-1 cells treated with DMSO (control), with Curcumin, for 24 h (Cur), and then released for 72 h (Rel). Graphs summarizing the results of three independent experiments. Un-paired *t*-test: *** *p* < 0.001, ** *p* < 0.01; (**C**) representative Giemsa-staining images of HCT116 cells with the indicated treatment. Scale bar = 10 μm. Graph summarizing the results of three independent experiments. Red arrows point to mitotic cells. Green arrows point to abnormal big nuclei. Un-paired *t*-test: * *p* < 0.05; (**D**) representative immunofluorescence images showing MPM2-positive HCT116 cells in the indicated treatments. Scale bar = 10 μm. Graph summarizing the results of three independent experiments. Un-paired *t*-test: * *p* < 0.05. (**E**). Graphs showing the percentage of SA-β-Gal-positive cells in the indicated cell lines and treatments. *n* = 3 (HCT116), *n* = 4 (RPE-1). Un-paired *t*-test: *** *p* < 0.001.

We treated HCT116 and RPE-1 cells with 10 μM curcumin for 24 h (Cur), and after a wash-out, cells were released in a fresh medium without the compound for an additional 72 h (release—Rel) (Figure 1A). According to Mosieniak et al. [18], this treatment induces senescence in HCT116 cells and other cancer cell lines; however, the effect on non-cancerous immortalized RPE-1 cells is not known. At the end of treatment, cells were collected, and flow cytometry was performed. The results showed that the cell cycle of RPE-1 cells was not compromised by curcumin administration (Figure 1B). On the contrary, HCT116 cells accumulated in the G2/M phase of the cell cycle after treatment with curcumin for 24 h, while they were mostly arrested in the G1 phase after 72 h of release (Figure 1B). A similar result was obtained with a different cancer cell line, MCF-7 cells (Supplementary Figure S1A).

To discriminate whether, following treatment with curcumin for 24 h, HCT116 cells were blocked in the G2 phase or mitosis, we stained the cells with Giemsa and evaluated the mitotic index (Figure 1C). These experiments clearly showed that curcumin-treated HCT116 cells (HCT116 Cur) accumulated in mitosis with respect to the control cells (HCT116 DMSO). On the contrary, the HCT116 cells released (HCT116 Rel) showed a decreased percentage of mitotic cells with respect to the control, as observed in MCF-7 cells, as well (Supplementary Figure S1A). We also confirmed these results via immunofluorescence analysis with the MPM2 antibody that detects a phospho-Ser/Thr-Pro epitope found in proteins phosphorylated mostly in mitosis (Figure 1D). Altogether, these results show that curcumin treatment for 24 h blocks HCT116 cells in mitosis and that after curcumin release, cells overcome the mitotic block, and eventually, arrest in the following G1 phase; this confirms that senescence might be induced. Furthermore, it is worth noting the presence of cells with large nuclei in the HCT116 Rel sample, which suggests polyploidy (Figure 1C). To directly evaluate the induced senescence in HCT116 and RPE-1 cells, we performed the Senescence Associated β-Galactosidase (SA-β-Gal) staining assay [29]. The obtained results confirmed the induction of senescence in HCT116 Rel cells [19] (Figure 1E). However, no significant induction of senescence was observed in RPE-1 cells following curcumin treatment and release (Figure 1E). Interestingly, senescence was also induced in MCF-7 cells released from curcumin (Supplementary Figure S1D). These experiments demonstrated that curcumin treatment selectively induces senescence in cancer cells, while it does not affect non-cancerous immortalized RPE-1 hTERT cells.

### 3.2. Curcumin Treatment Induces Mitotic Slippage and DNA Damage in Cancer Cells

**Figure 2:**
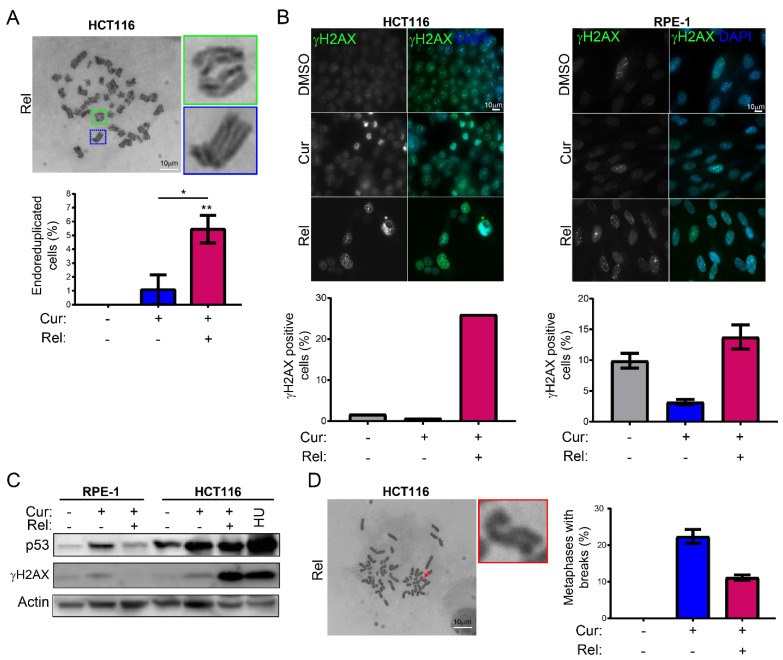
Curcumin treatment induces mitotic slippage and DNA damage: (**A**) Representative Giemsa-staining images of metaphase spreads of HCT116 cells with the indicated treatment. Enlargements of few chromosomes are attached on the right. Scale bar = 10 μm. Graph showing the percentage of endoreduplicated cells for each indicated sample. *n* = 3. Un-paired *t*-test: ** *p* < 0.01, * *p* < 0.05; (**B**) representative immunofluorescence images showing gH2AX-positive HCT116 and RPE-1 cells in the indicated cell treatment. Scale bar = 10 μm. Graph summarizing the results of one experiment for HCT116 cells and two independent experiments for RPE-1 cells; (**C**) immunoblots of cell extracts with antibodies against p53 and γH2AX. HCT116 cells treated with hydroxyurea (HU) are used as a positive control for DNA damage. Antibody against actin is used as a loading control; (**D**) Representative metaphase spread image showing chromosome break in HCT116 cells with the indicated cell treatment. Arrows indicate chromosome. On the right side, there is an enlargement of a broken chromosome. Scale bar = 10 μm. Graph summarizing the results of two independent experiments.

Afterwards, we investigated the possible reason(s) for the triggering of senescence in HCT116 cells following curcumin treatment and release. Since it has been demonstrated that cell senescence can be induced by mitotic slippage [30,31], we evaluated, in HCT116 Rel cells, the presence of tetraploid/polyploid cells, which are suggestive of mitotic slippage. To this aim, we looked at the metaphases of HCT116 Cur and Rel cells treated with Colcemid or left untreated. Interestingly, the results show that about 5.4% of observed metaphases are endoreduplicated in the HCT116 Rel sample (Figure 2A). Moreover, Giemsa staining also showed the presence of abnormal big nuclei (Figure 1C), suggesting a polyploid content, which is in accordance with mitotic slippage. Mitotic slippage can also go along with DNA damage, which, in turn, could contribute to senescence induction. Immunofluorescence and immunoblotting targeting γH2AX showed the occurrence of DNA damage in HCT116 Rel cells with respect to control (Figure 2B-C). On the contrary, DNA damage levels were not significantly different between RPE-1 DMSO, Cur, and Rel cells (Figure 2B,C). The activation of the DNA damage response was assessed via immunoblotting against p53, which showed an increase in the protein in HCT116 Rel cells, and also in both HCT116 and RPE-1 cells, after 24h of curcumin treatment (Figure 2C). In addition, we observed an increase in metaphases with chromosome breaks in both Cur and Rel HCT116 cells (Figure 2D).

These observations suggest that curcumin treatment could induce senescence in HCT116 cells by causing mitotic slippage and DNA damage.

### 3.3. Curcumin-Induced Senescence Is Associated with p21^waf1/cip1^ Activation and Global Reduction in Heterochromatin Markers

**Figure 3:**
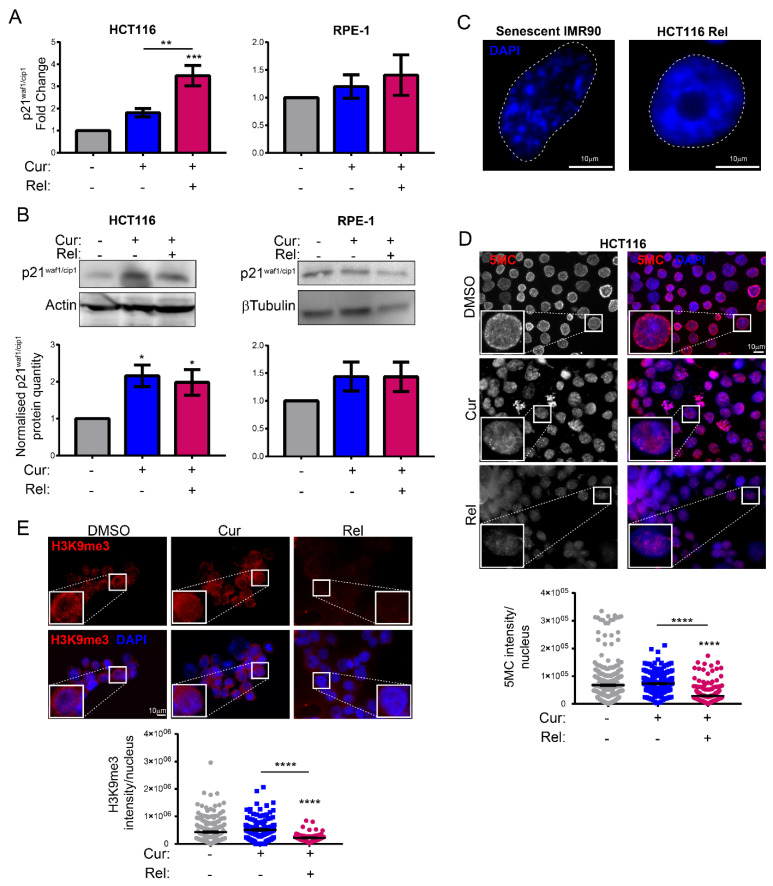
Curcumin-induced senescence correlates with p21 activation and global reduction in heterochromatin markers: (**A**) Histograms showing the expression of the p21 gene in HCT116/RPE-1 Cur and Rel cells relative to control. *n* = 3. Un-paired *t*-test: *** *p* < 0.001, ** *p* < 0.01; (**B**) immunoblots of cell extracts with antibodies against p21. Antibodies against β-tubulin or actin are used as a loading control. Graphs summarizing the quantification of three independent experiments. Un-paired *t*-test: * *p* < 0.05; (**C**) representative DAPI-staining images of senescent IMR90 cells and HCT116 Rel cells. SAHF are visible only in senescent IMR90 nuclei, which are used as a control. *n* = 2. Scale bar = 10 μm; (**D**) representative immunofluorescence images showing the pattern of 5-methyl-cytosine (5MC) antibodies in HCT116 cells with the indicated treatment. Scale bar = 10 μm. Graph showing the global reduction in 5MC in HCT116 Rel cells. *n* = 3. Un-paired *t*-test: **** *p* < 0.0001; (**E**) representative immunofluorescence images showing the pattern of H3K9me3 antibodies in HCT116 cells with the indicated treatment. Scale bar = 10 μm. Graph showing the global reduction in H3K9me3 in HCT116 Rel cells. *n* = 3. Un-paired *t*-test: **** *p* < 0.0001.

To further characterize curcumin-induced senescence, we evaluated the presence of senescence-associated markers. Then, we investigated the increase in p21^waf1/cip1^, which is largely considered responsible for the permanent cell-cycle arrest of senescent cells, together with p16^ink4a^ [15]. The results show that both the transcript and the p21^waf1/cip1^ protein were increased in HCT116 Cur and Rel cells with respect to control cells (Figure 3A,B). An increase in p21^waf1/cip1^ was also observed in MCF-7 cells under the same treatment (Supplementary Figure S1B,C). Additionally, p16^Ink4a^ was not increased in HCT116 cells (data not shown), suggesting that the curcumin-induced senescence was p21^waf1/cip1^-dependent. Importantly, p21^waf1/cip1^ was significantly increased neither in RPE-1 Cur nor in RPE-1 Rel with respect to control cells (Figure 3A,B), confirming that neither cell-cycle arrest nor cell senescence occur in non-cancerous immortalized cells under curcumin treatment.

Apart from SA-β-Gal activity, senescent cells are often characterized by SAHF. Thus, we stained formaldehyde-fixed nuclei with DAPI, and did not observe SAHF in any of the nuclei of HCT116 Rel cells to be different from senescent IMR90 nuclei—herein used as a control—which showed the typical DAPI-stained foci (SAHF) (Figure 3C). This suggests that curcumin-induced senescence is not associated with SAHF formation.

Since senescent cells undergo other chromatin rearrangements, mainly global heterochromatin loss, we evaluated two important markers of heterochromatin. Firstly, we checked the levels of global DNA methylation in HCT116 control, Cur, and Rel cells via immunofluorescence against 5-Methylcytosine (5MC). The results showed that HCT116 Rel cells had a significantly lower nuclear level of 5MC compared to the control cells (Figure 3D). On the contrary, DNA methylation levels in HCT116 Cur nuclei did not change with respect to the control cells (Figure 3D). Moreover, we noticed a different pattern of 5MC staining in the nuclei, mostly occurring at the nuclear periphery in control cells and randomly distributed in the nucleoplasm in both HCT116 Cur and Rel cells (Figure 3D). Then, we tested the levels of H3K9 trimethylation (H3K9me3), another marker of heterochromatin. Immunofluorescence images showed that, similarly to 5-Methylcytosine, H3K9me3 was globally reduced in the nucleus in HCT116 Rel cells with respect to the control (Figure 3E).

Altogether these results demonstrate that curcumin-induced senescence in HCT116 cells is characterized by typical senescence markers—namely p21^waf1/cip1^ activation, SA-β-Gal activity and the global loss of heterochromatin markers (DNA methylation and H3K9me3)—while it is not associated with SAHF.

### 3.4. Curcumin-Induced Senescent Cancer Cells Are Cleared via Treatment with Senolytics

**Figure 4:**
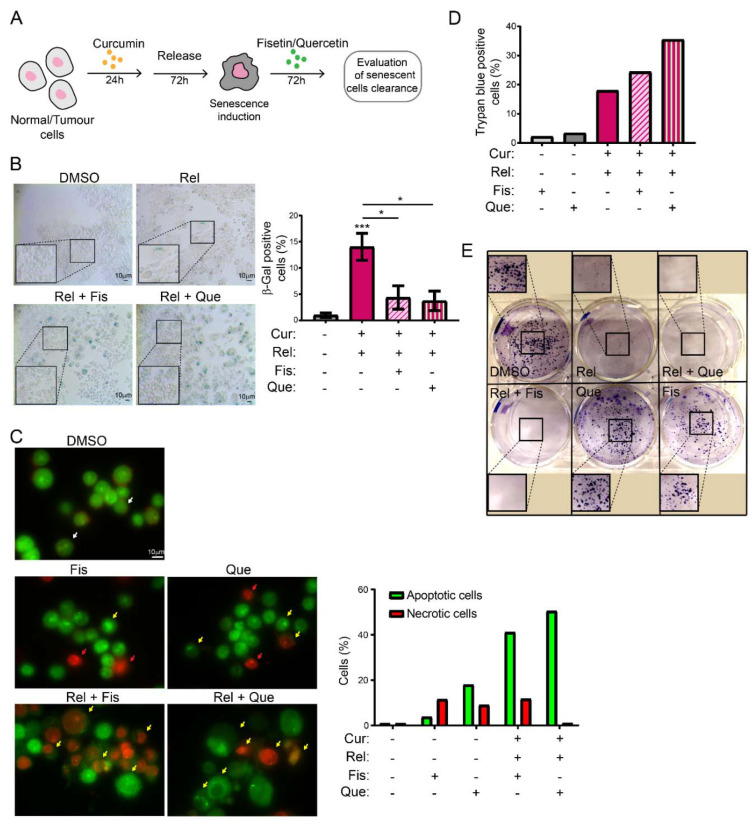
Curcumin-induced senescent cancer cells are cleared by treatment with senolytic drugs (**A**) Schematics of the experiment workflow; (**B**) representative images of SA-β-GAL staining in HCT116 cells after curcumin release and subsequent treatment for 72 h with Fisetin or Quercetin. On the lower-left corner of each image, there is an enlargement of a part of the same image. Graph summarizing the results of two independent experiments with two technical replicates each. Un-paired *t*-test: *** *p* < 0.001, * *p* < 0.05; (**C**) AO/EB double-staining assay for apoptosis detection shows an increase in apoptotic cells following senolytic treatments in curcumin-treated cells. Apoptotic cells (yellow arrow) show membrane blebs and apoptotic vesicles, and fragmented chromatin appears in red following ethidium bromide staining that enters cells when plasma membrane is damaged (necrosis—red arrow). Living cells are uniformly stained in green (white arrow). Scale bar= 10 μm; (**D**) Trypan Blue viability assay showing increased mortality following the double treatments of curcumin + senolytics; (**E**) colony assay demonstrates the impaired proliferation of curcumin-treated cells following senolytics administration.

Once we assessed the specific senescence induction in curcumin-treated cancer cells, we then tested whether curcumin-induced senescent cancer cells could be targeted by natural compounds, whose senolytic activity had been assessed. To this aim, we treated HCT116 cells released from curcumin treatment with fisetin or quercetin for an additional 72 h (Figure 4A). To avoid any side-effects related to the modest pleiotropic action of senolytics, we used a concentration of 5 µM for both fisetin (Fis) and quercetin (Que), which has neither cytotoxic nor anti-proliferative effects on non-cancerous immortalized cells (Supplementary Figure S2). Then, we evaluated whether the combined treatments with curcumin reduced the number of senescent HCT116 Rel cells. The results showed that the percentage of SA-β-Gal positive cells decreased in HCT116 Rel + Fis and Rel + Que cells compared to HCT116 Rel cells, and that this percentage dropped to levels whose tendency was close to that of the control cells (Figure 4B). Moreover, AO/EB staining showed an increased apoptotic response following treatment with both fisetin and quercetin, especially in curcumin-treated cells. The control displayed only a small number of necrotic cells. Cell death in HCT116 Rel + Fis and Rel + Que cells was also confirmed via the Trypan Blue assay. Moreover, we performed a clonogenic assay with crystal violet staining, which clearly indicated that cells treated with both curcumin and senolytics do not grow. These results suggest that the combination of curcumin and fisetin/quercetin could be exploited as a cancer therapy.

## 4. Discussion

Cell senescence can be induced by a plethora of stressors and stimuli, including telomere shortening (replicative senescence), oncogene activation (oncogene-induced senescence, OIS), mitochondrial stress, cell fusions, oxidative stress, and inflammatory cytokines [32]. Most of these lead to DNA damage, which, in turn, triggers permanent cell-cycle arrest (senescence). In the most recent years, it has been also demonstrated that mitotic stress can promote cell-cycle arrest of OIS [30]. Extended mitotic arrest could result in either abnormal cell division with aneuploidy generation after the arrest, in mitotic catastrophe and cell death, or in mitotic slippage whereby cells bypass cell division, returning to interphase with tetraploid DNA content (reviewed in [33]). Then, the mitosis-bypassing cells undergo cell death or cell senescence after slippage [31,34,35,36]. If the cells survive the mitotic slippage, endoreduplication usually occurs, and cells become giant and accumulate DNA damage [31,37]. All of this triggers the transition to cell senescence. Here, we show that a similar fate characterizes cancer cells treated with curcumin. Curcumin shows an elevated degree of pleiotropy, which is responsible for the heterogeneous responses observed following its administration. Furthermore, curcumin triggers the downregulation of many genes involved in the inflammatory response, displaying anti-inflammatory activity, as well as the downregulation of anti-apoptotic proteins [20,21]. Lastly, it has been related to the HAT p300 inhibition and to a reduction in CDK1 transcription, which probably underlies the G2/M arrest observed in cancer cells [38].

We observed that curcumin first induces selective cell-cycle arrest in mitosis only in cancer cells, and not in non-cancerous immortalized cells. When cancer cells are then released from curcumin, they can escape mitotic arrest, undergoing mitotic slippage and, partly, endoreduplication. In fact, in cancer cells, we observed the appearance of endoreduplicated chromosomes (Figure 2A) and big nuclei suggestive of a hyperdiploid content. We also noticed that the escaped cells accumulate chromosome breaks and DNA damage (γH2AX foci and protein increase) (Figure 2B–D). Curiously, curcumin treatment for 24 h induced an increase in the tumor suppressor p53 in both HCT116 and RPE-1 cells, suggesting the activation of the DNA damage checkpoint. However, while RPE-1 cells fully recovered during curcumin release (a reduction to normal levels of p53 and γH2AX proteins, Figure 2C), HCT116 cells underwent a further increase in γH2AX and p53, suggesting that in this cancer-cell line, curcumin insult cannot be fixed. All of this probably induces the observed senescence in cancer cells. Senescence induction, as previously reported [18], was, indeed, supported by SA-β-Gal activity, which highly increased, specifically in curcumin-released cancer cells, compared to the untreated condition (Figure 1E). Senescence induction was also suggested by the larger and flatter morphology observed in the cells released from the treatment. The result is particularly striking when compared to the non-cancerous immortalized cells, which display no significant increase in SA-β-Gal activity upon release from curcumin treatment (Figure 1E). This finding suggests the selectivity of curcumin action towards cancer cells. We show that curcumin-induced senescence depends on p21^waf1/cip1^ activation, which further confirmed the upstream activation of p53. We also demonstrate that curcumin-induced senescent cancer cells undergo global heterochromatin loss, assessed by the nuclear reduction in the heterochromatin markers DNA methylation (5MC) and H3K9me3. Moreover, the localization of the staining of these markers in the nuclei changes, moving away from the nuclear periphery towards the nucleoplasm. This matches with the epigenetic rearrangements that cells experience during senescence [13]. However, we have not observed SAHF in curcumin-induced senescent cancer cells, which is similar to what was observed in other senescence contexts, and which could reinforce the hypothesis that SAHF formation is a two-step mechanism [17]. We tested senescence markers of the early phases of senescence establishment that are not compromised by the side effects of flavonoids. On the other hand, SASP is an important feature of senescent cells, at least in overt senescent cells. However, in this case, SASP might not be involved, because of the well-known curcumin function in the de-regulation of genes related to the inflammatory response. It has been shown that curcumin strongly reduces NF-κB and IL-1α expression [39,40], which are the main protein factors responsible for SASP initiation and maintenance. However, SASP occurrence could be tested to evaluate its cause–effect relationship with a persistent and transient DNA damage response [41,42].

Selectively inducing senescence only in cancer cells and not in non-cancerous immortalized cells could be, by itself, an interesting strategy to block cancer cells’ proliferation in patients. However, the persistence of a therapy that induces senescence could be detrimental in the long term. Thus, the research of strategies aimed to eliminate therapy-induced senescent cells has recently acquired interest. This would minimize both the collateral effect of long-term treatment with senescence-inducing activity, and the possibility of tumor resumption. There are mainly two lines of research in this context: targeting senescent cancer cells with senolytic drugs or with senolysis mediated by the immune response (reviewed in [43]). Thus, we explored the possibility of combining curcumin treatment with the administration of other natural compounds with senolytic activity, in an attempt to clear curcumin-induced senescent cells. The obtained results suggest that fisetin and quercetin reduce the number of curcumin-induced senescent cells by inducing an apoptotic response, which is coherent with the current knowledge of senolytics’ action. In fact, AO/EB staining revealed cells whose morphology, following curcumin and senolytic treatments, was dramatically altered, with membrane blebs and chromatin fragmentations which are typical of apoptosis. The bright green and yellow staining of cells treated with curcumin and senolytics, as well as the red staining of nuclei and chromatin, reinforces the assumption that apoptosis was induced, since these features are suggestive of early and late apoptosis, respectively. Ethidium bromide can only stain DNA red if the plasma membrane integrity is lost, a phenomenon that occurs only during apoptosis or necrosis. However, this last outcome can easily be excluded, since the green staining (which is typical of viable or early apoptotic cells), membrane blebs and apoptotic vesicles are also detectable. In parallel, the number of non-cancerous, not-senescent, proliferating cells increases following treatment with senolytics, meaning that their action is specific and does not affect non-cancerous immortalized cells. On the contrary, in cancer cells, viability assays displayed a cytotoxic response, as well as an impairment of cell proliferation, following curcumin + senolytics treatments. This outcome, combined with the general anti-inflammatory action of flavonoids, represents a potential strategy which can be safer for patients, given that a chronic inflammatory state has often been related to enhanced tumorigenesis. Our observations, thus, pave the way to a promising therapeutical strategy against cancer, based on natural compounds.

## Data Availability

Data are contained within the article or supplementary material. Images of all Western blots, immunofluorescence, and metaphase spreads are available upon request from the corresponding author.

## Supplementary Materials

The following supporting information can be downloaded at: https://www.mdpi.com/article/10.3390/genes13071125/s1, Figure S1: Curcumin triggers senescence induction in MCF-7 cells through cell-cycle arrest and in a p21^waf1/cip1^ -dependent manner: (A) Representative flow-cytometric profiles showing the distribution throughout the cell cycle of propidium iodide-labeled MCF-7 cells following Curcumin treatment for 24 h, and then released for 72 h. DMSO was used for the control condition. *n* = 2; (B) RT-qPCR showing p21^waf1/cip1^ increase after 72 h of release from Curcumin treatment. *n* = 1; (C) immunoblotting quantification shows the increase in p21^waf1/cip1^ protein levels after 24 h of treatment of Curcumin and 72 h of release from the treatment. *n* = 1; (D) SA-β-Gal assay showing the increase in the percentage of blue-stained positive cells following 72 h of release from Curcumin treatment. *n* = 2. Figure S2: Senolytic drugs Quercetin and Fisetin show no cytotoxic or antiproliferative effects at the concentrations 5 and 10 μM: (A) Trypan Blue assay showed no toxicity in senolytic-treated RPE-1 hTERT and HCT116 cells at the 5 and 10 μM doses; (B) representative flow-cytometric profiles showing the distribution throughout the cell cycle of propidium iodide-labeled HCT116 cells, following quercetin treatment for 72 h at different concentrations. The molecule does not halt cell proliferation or show any anti-proliferative activity.

## References

[1] Ozaki T., Nakagawara A. (2011). Role of P53 in Cell Death and Human Cancers. Cancers.

[2] Hayflick L., Moorhead P.S. (1961). The Serial Cultivation of Human Diploid Cell Strains. Exp. Cell Res..

[3] D’Adda di Fagagna F., Reaper P.M., Clay-Farrace L., Fiegler H., Carr P., Von Zglinicki T., Saretzki G., Carter N.P., Jackson S.P. (2003). A DNA Damage Checkpoint Response in Telomere-Initiated Senescence. Nature.

[4] Fumagalli M., Rossiello F., Clerici M., Barozzi S., Cittaro D., Kaplunov J.M., Bucci G., Dobreva M., Matti V., Beausejour C.M. (2012). Telomeric DNA Damage Is Irreparable and Causes Persistent DNA-Damage-Response Activation. Nat. Cell Biol..

[5] Di Micco R., Krizhanovsky V., Baker D., d’Adda di Fagagna F. (2021). Cellular Senescence in Ageing: From Mechanisms to Therapeutic Opportunities. Nat. Rev. Mol. Cell Biol..

[6] Carnero A., Galluzzi L., Vitale I., Kepp O., Kroemer G. (2013). Markers of Cellular Senescence. Cell Senescence: Methods and Protocols.

[7] Salmonowicz H., Passos J.F. (2017). Detecting Senescence: A New Method for an Old Pigment. Aging Cell.

[8] Acosta J.C., Banito A., Wuestefeld T., Georgilis A., Janich P., Morton J.P., Athineos D., Kang T.-W., Lasitschka F., Andrulis M. (2013). A Complex Secretory Program Orchestrated by the Inflammasome Controls Paracrine Senescence. Nat. Cell Biol..

[9] González-Gualda E., Baker A.G., Fruk L., Muñoz-Espín D. (2021). A Guide to Assessing Cellular Senescence In Vitro and In Vivo. FEBS J..

[10] Pappalardo X.G., Barra V. (2021). Losing DNA Methylation at Repetitive Elements and Breaking Bad. Epigenet. Chromatin.

[11] Narita M., Nuñez S., Heard E., Narita M., Lin A.W., Hearn S.A., Spector D.L., Hannon G.J., Lowe S.W. (2003). Rb-Mediated Heterochromatin Formation and Silencing of E2F Target Genes during Cellular Senescence. Cell.

[12] Chandra T., Kirschner K., Thuret J.-Y., Pope B.D., Ryba T., Newman S., Ahmed K., Samarajiwa S.A., Salama R., Carroll T. (2012). Independence of Repressive Histone Marks and Chromatin Compaction during Senescent Heterochromatic Layer Formation. Mol. Cell.

[13] Sidler C., Kovalchuk O., Kovalchuk I. (2017). Epigenetic Regulation of Cellular Senescence and Aging. Front. Genet..

[14] Paluvai H., Di Giorgio E., Brancolini C. (2020). The Histone Code of Senescence. Cells.

[15] Kumari R., Jat P. (2021). Mechanisms of Cellular Senescence: Cell Cycle Arrest and Senescence Associated Secretory Phenotype. Front. Cell Dev. Biol..

[16] Kosar M., Bartkova J., Hubackova S., Hodny Z., Lukas J., Bartek J. (2011). Senescence-Associated Heterochromatin Foci Are Dispensable for Cellular Senescence, Occur in a Cell Type- and Insult-Dependent Manner and Follow Expression of P16 (Ink4a). Cell Cycle Georget. Tex.

[17] Chandra T., Ewels P.A., Schoenfelder S., Furlan-Magaril M., Wingett S.W., Kirschner K., Thuret J.-Y., Andrews S., Fraser P., Reik W. (2015). Global Reorganization of the Nuclear Landscape in Senescent Cells. Cell Rep..

[18] Mosieniak G., Adamowicz M., Alster O., Jaskowiak H., Szczepankiewicz A.A., Wilczynski G.M., Ciechomska I.A., Sikora E. (2012). Curcumin Induces Permanent Growth Arrest of Human Colon Cancer Cells: Link between Senescence and Autophagy. Mech. Ageing Dev..

[19] Hatamipour M., Johnston T.P., Sahebkar A. (2018). One Molecule, Many Targets and Numerous Effects: The Pleiotropy of Curcumin Lies in Its Chemical Structure. Curr. Pharm. Des..

[20] Liao S.-C., Hsu H.-W., Chuang K.-L., Huang Z.-Y., Lin K.-T., Hsu W.-H., Chang K.-H., Huang C.-Y.F., Su C.-L. (2019). Using the Pleiotropic Characteristics of Curcumin to Validate the Potential Application of a Novel Gene Expression Screening Platform. Nutrients.

[21] Hewlings S.J., Kalman D.S. (2017). Curcumin: A Review of Its’ Effects on Human Health. Foods.

[22] Malavolta M., Bracci M., Santarelli L., Sayeed M.A., Pierpaoli E., Giacconi R., Costarelli L., Piacenza F., Basso A., Cardelli M. (2018). Inducers of Senescence, Toxic Compounds, and Senolytics: The Multiple Faces of Nrf2-Activating Phytochemicals in Cancer Adjuvant Therapy. Mediat. Inflamm..

[23] Rolt A., Cox L.S. (2020). Structural Basis of the Anti-Ageing Effects of Polyphenolics: Mitigation of Oxidative Stress. BMC Chem..

[24] Domaszewska-Szostek A., Puzianowska-Kuźnicka M., Kuryłowicz A. (2021). Flavonoids in Skin Senescence Prevention and Treatment. Int. J. Mol. Sci..

[25] Massaro M., Barone G., Barra V., Cancemi P., Di Leonardo A., Grossi G., Lo Celso F., Schenone S., Viseras Iborra C., Riela S. (2021). Pyrazole[3,4-d]Pyrimidine Derivatives Loaded into Halloysite as Potential CDK Inhibitors. Int. J. Pharm..

[26] Fragkos M., Barra V., Egger T., Bordignon B., Lemacon D., Naim V., Coquelle A. (2019). Dicer Prevents Genome Instability in Response to Replication Stress. Oncotarget.

[27] Fernandes P., Miotto B., Saint-Ruf C., Said M., Barra V., Nähse V., Ravera S., Cappelli E., Naim V. (2021). FANCD2 Modulates the Mitochondrial Stress Response to Prevent Common Fragile Site Instability. Commun. Biol..

[28] Aird K.M., Zhang R. (2013). Detection of Senescence-Associated Heterochromatin Foci (SAHF). Methods Mol. Biol. Clifton NJ.

[29] Dimri G.P., Lee X., Basile G., Acosta M., Scott G., Roskelley C., Medrano E.E., Linskens M., Rubelj I., Pereira-Smith O. (1995). A Biomarker That Identifies Senescent Human Cells in Culture and in Aging Skin in Vivo. Proc. Natl. Acad. Sci. USA.

[30] Dikovskaya D., Cole J.J., Mason S.M., Nixon C., Karim S.A., McGarry L., Clark W., Hewitt R.N., Sammons M.A., Zhu J. (2015). Mitotic Stress Is an Integral Part of the Oncogene-Induced Senescence Program That Promotes Multinucleation and Cell Cycle Arrest. Cell Rep..

[31] Tsuda Y., Iimori M., Nakashima Y., Nakanishi R., Ando K., Ohgaki K., Kitao H., Saeki H., Oki E., Maehara Y. (2017). Mitotic Slippage and the Subsequent Cell Fates after Inhibition of Aurora B during Tubulin-Binding Agent-Induced Mitotic Arrest. Sci. Rep..

[32] Martínez-Zamudio R.I., Robinson L., Roux P.-F., Bischof O. (2017). SnapShot: Cellular Senescence Pathways. Cell.

[33] Ghelli Luserna di Rorà A., Martinelli G., Simonetti G. (2019). The Balance between Mitotic Death and Mitotic Slippage in Acute Leukemia: A New Therapeutic Window?. J. Hematol. Oncol. J. Hematol. Oncol..

[34] Cheng B., Crasta K. (2017). Consequences of Mitotic Slippage for Antimicrotubule Drug Therapy. Endocr. Relat. Cancer.

[35] Balachandran R.S., Kipreos E.T. (2017). Addressing a Weakness of Anticancer Therapy with Mitosis Inhibitors: Mitotic Slippage. Mol. Cell. Oncol..

[36] Jakhar R., Luijten M.N.H., Wong A.X.F., Cheng B., Guo K., Neo S.P., Au B., Kulkarni M., Lim K.J., Maimaiti J. (2018). Autophagy Governs Protumorigenic Effects of Mitotic Slippage-Induced Senescence. Mol. Cancer Res. MCR.

[37] Orth J.D., Loewer A., Lahav G., Mitchison T.J. (2012). Prolonged Mitotic Arrest Triggers Partial Activation of Apoptosis, Resulting in DNA Damage and P53 Induction. Mol. Biol. Cell.

[38] Prieur A., Besnard E., Babled A., Lemaitre J.-M. (2011). P53 and P16INK4A Independent Induction of Senescence by Chromatin-Dependent Alteration of S-Phase Progression. Nat. Commun..

[39] Das L., Vinayak M. (2014). Curcumin attenuates carcinogenesis by down regulating proinflammatory cytokine interleukin-1 (IL-1α and IL-1β) via modulation of AP-1 and NF-IL6 in lymphoma bearing mice. Int Immunopharmacol..

[40] Olivera A., Moore T.W., Hu F., Brown A.P., Sun A., Liotta D.C., Snyder J.P., Yoon Y., Shim H., Marcus A.I. (2012). Inhibition of the NF-κB signaling pathway by the curcumin analog, 3,5-Bis(2-pyridinylmethylidene)-4-piperidone (EF31): Anti-inflammatory and anti-cancer properties. Int. Immunopharmacol..

[41] Rodier F., Coppé J.P., Patil C.K., Hoeijmakers W.A., Muñoz D.P., Raza S.R., Freund A., Campeau E., Davalos A.R., Campisi J. (2009). Persistent DNA damage signalling triggers senescence-associated inflammatory cytokine secretion. Nat. Cell Biol..

[42] Cuollo L., Antonangeli F., Santoni A., Soriani A. (2020). The Senescence-Associated Secretory Phenotype (SASP) in the Challenging Future of Cancer Therapy and Age-Related Diseases. Biology.

[43] Wang L., Lankhorst L., Bernards R. (2022). Exploiting Senescence for the Treatment of Cancer. Nat. Rev. Cancer.

