# Supplementary material for: Specific Irreversible Cell-Cycle Arrest and Depletion of Cancer Cells Obtained by Combining Curcumin and the Flavonoids Quercetin and Fisetin
Source: Genes (Basel). 2022 Jun 23;13(7):1125. doi: 10.3390/genes13071125 (PMC9316914; doi:10.3390/genes13071125)
Supplement: Supplementary file 1 [file genes-13-01125-s001.zip › genes-1732116-supplementary.pdf]

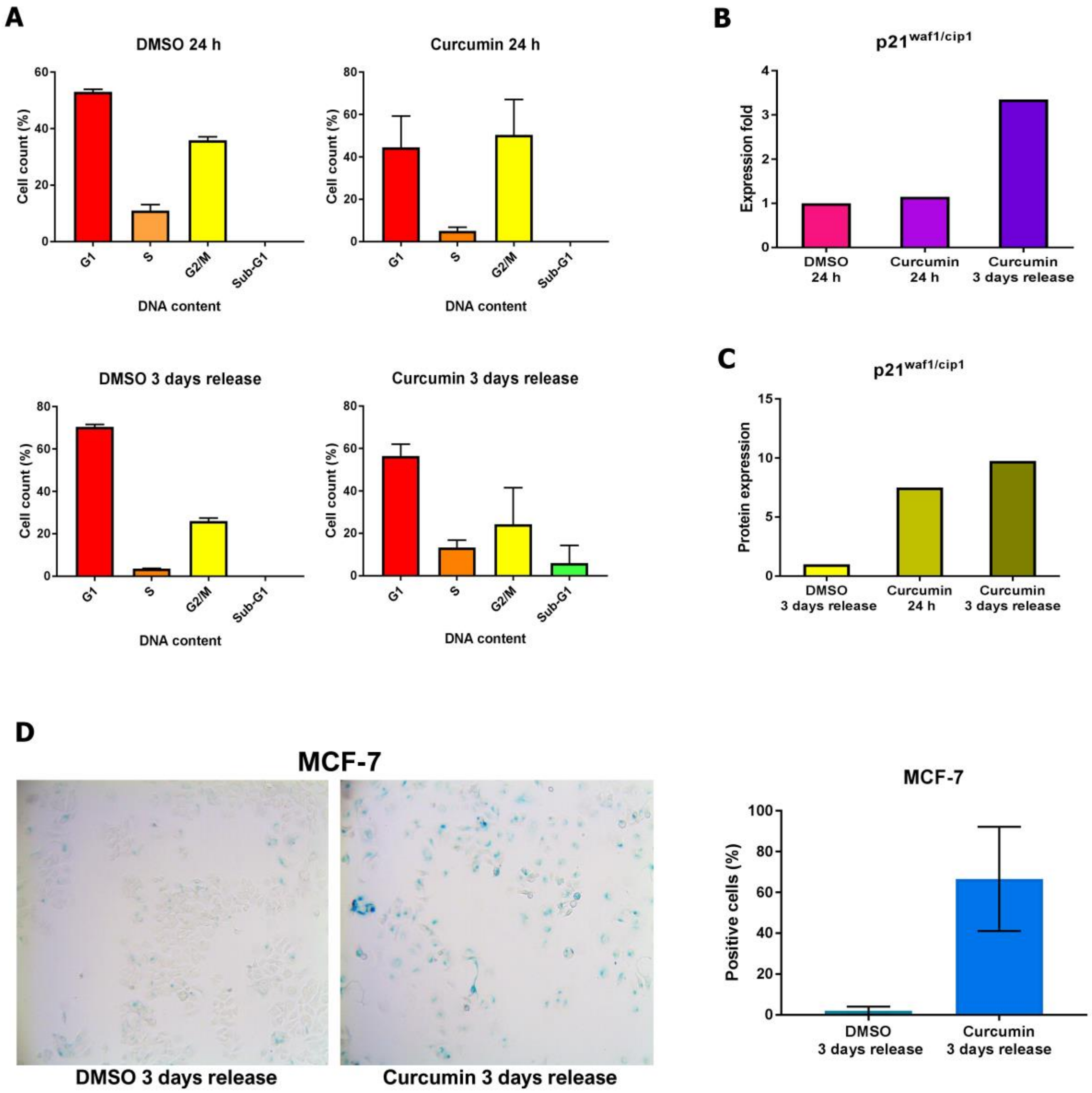

Figure S1. Curcumin triggers senescence induction in MCF-7 cells through cell-cycle arrest and in a p21$^{waf1/cip1}$ - dependent manner.

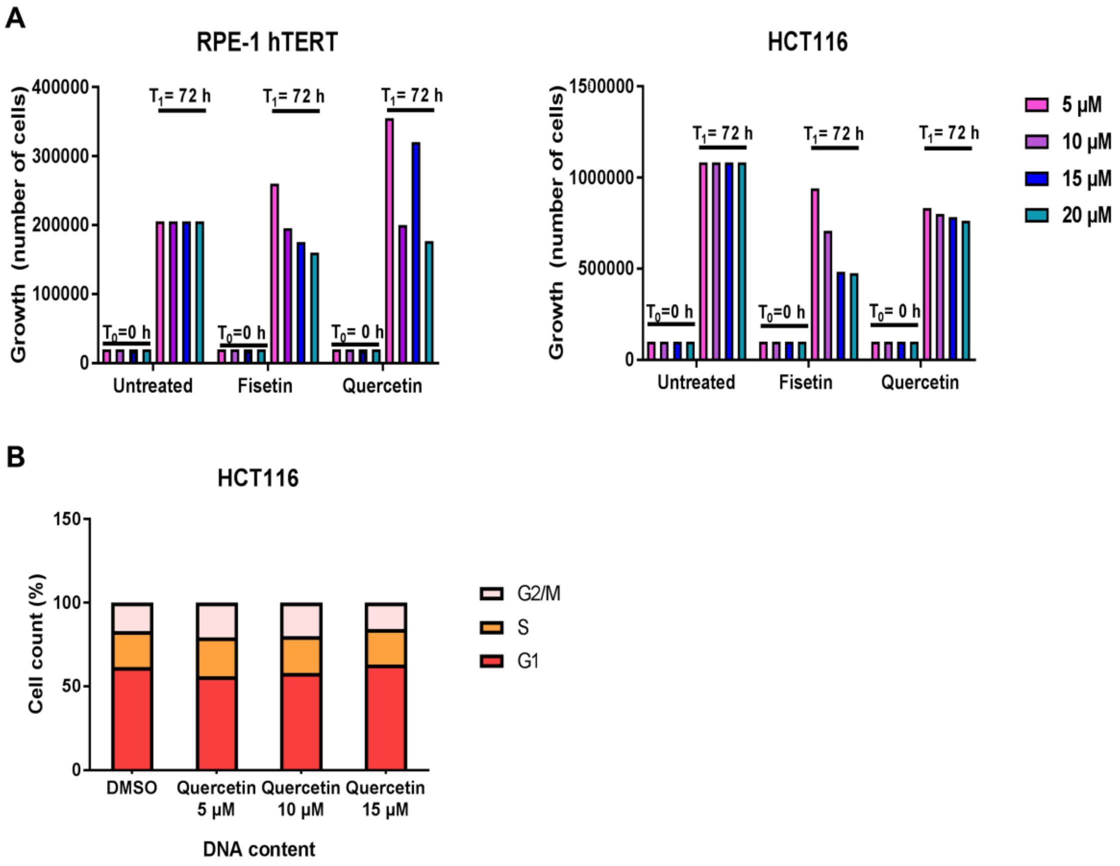

Figure S2: Senolytic drugs Quercetin and Fisetin show no cytotoxic or antiproliferative effects at the concentrations 5 and 10 µM.
